# PReS-FINAL-2323: Cryoglobulinemic vasculitis preceding diagnosis of Carney complex

**DOI:** 10.1186/1546-0096-11-S2-P313

**Published:** 2013-12-05

**Authors:** MB Glerup, C Heuck, J Bjerre, T Herlin

**Affiliations:** 1 Pediatrics, Aarhus University Hospital, Skejby, Aarhus, Denmark

## Introduction

Carney complex (CNC) is a disorder characterized by skin pigmentary abnormalities and benign cardiac,endocrine, skin and neuronal tumors. Areas of unusual lentigines are the most common presenting feature of CNC usually around the lips, eyes or genitalia increase in number at puberty. Cardiac myxomas may occur in any or all cardiac chambers, and leading to intracardiac obstruction of blood flow, embolic phenomena, and/or heart failure. Carney complex is a rare autosomal dominant disease. The complex has been mapped to 2p16 (CNC2) and 17q22-24 (CNC1), and mutations in the tumor suppressor gene protein kinase A regulatory subunit 1-alpha, *PRKAR1A*, are causative [1].

## Objectives

A previously healthy 12-year-old boy presented with recurrent pain and swelling initially in the left foot later followed by the right one. The swelling regressed spontaneously over weeks but the pain remained. At an age of 13 years the patient had stiffness of the fingers on the right hand and the toes were painfull but without swelling. He had no fever. At first presentation erythrocyte sedimentation rate (ESR) was elevated to 34 mm/h, CRP was normal. Furthermore, he had thrombocytosis (500 x 109/L), anemia (10.5 g/dL), IgM slightly elevated, normal complement C3c and C4. Rheumatoid factor, ANCA and ANA were all negative but cryoglobulins (not further specified) were positive. An unusual pigmentation around the lips were noticed. He had monthly attacks lasting 5-10 days with pain and swelling of the feet well treated with Prednisolone and Azathioprine. 2 years later he was admitted with confusion, aphasia and apraxia but without seizures. The symptoms regressed spontaneously after a few hours.

## Methods

MRI was performed with a 1.5 Tesla scanning system including T1, T2, T2-flair and diffusion-weighted sequences as well as MR-angiography using 3-dimensional time of flight. Genetic testing of *PRKAR1A *gene mutation analysis was done by conventional techniques.

## Results

MR scans revealed parieto-occipital infarction but no signs of schwannomas or vasculitis on the angiography. An echocardiography revealed an obstructive tumor, 5-6 centimeters in diameter, in the left atrium, which was surgically removed.The lentigines and the myxoma led to the suggestion of Carney Complex. Diagnosis was confirmed by a *PRKAR1A *mutation found in the child as well as the mother.

## Conclusion

To our knowledge, this is the first case of combined Carney Complex and cryoglobulinemia. A few cases of vasculitis and CNC have been reported, which makes it likely to assume a connection. We suggest that vasculitis should be added to the list of presenting symptoms of CNC. A diagnosis of CNC should be considered in patients who present with vasculitis, unusual lentigines, and myxomas, endocrine tumors or schwannomas.

## Disclosure of interest

None declared.
